# Does the expression of the *ACVR2A* gene affect the development of colorectal cancer?

**DOI:** 10.1590/1678-4685-GMB-2017-0332

**Published:** 2019-03-11

**Authors:** Damian Wodziński, Agnieszka Wosiak, Jacek Pietrzak, Rafał Świechowski, Agnieszka Jeleń, Ewa Balcerczak

**Affiliations:** 1 Medical University of Lodz Medical University of Lodz Department of Pharmaceutical Biochemistry and Molecular Diagnostics, Interfaculty Cathedral of Laboratory and Molecular Diagnostics Department of Pharmaceutical Biochemistry and Molecular Diagnostics, Interfaculty Cathedral of Laboratory and Molecular Diagnostics Lodz Poland Laboratory of Molecular Diagnostics and Pharmacogenomics, Department of Pharmaceutical Biochemistry and Molecular Diagnostics, Interfaculty Cathedral of Laboratory and Molecular Diagnostics, Medical University of Lodz, Lodz, Poland

**Keywords:** Activin A protein, *ACVR2A* gene expression, tumor suppressor, colorectal cancer, TGFß signaling pathway

## Abstract

Colorectal cancer has become a serious problem, especially in highly developed countries. As reported by the World Health Organization, the number of colon cancer cases in the world in 2012 amounted to 1.36 million. It is the second most common cancer in females (614,000 cases, 9.2% of the total) and the third in males (746,000 cases, 10.0% of the total) worldwide. It is believed that TGFβ pathway elements are involved in the pathogenesis of colorectal cancer. This study assessed one of these elements, the *ACVR2A* gene. Qualitative and quantitative analyses of the *ACVR2A* gene in 84 patients with colorectal cancer was performed. There was no statistically significant association between *ACVR2A* gene expression and age, gender, histological type, grading of tumor, vascular invasion, and presence of lymphocytes in tumor tissue. No association was observed between the *ACVR2A* gene expression level and the presence of metastases in regional lymph nodes and distant metastases. In this study, larger tumors (T3 and T4) were characterized by higher *ACVR2A* expression compared to smaller tumors (T1 and T2). This may indicate an association between *ACVR2A* expression and the severity of pathological changes in the tumor growth process.

## Introduction

Risk factors for colorectal cancer can be divided into modifiable and non-modifiable types. The former one includes bad nutritional habits, physical inactivity, obesity, cigarette smoking, heavy alcohol consumption, and urban residence. The non-modifiable risk factors include age and hereditary factors. The likelihood of colorectal cancer diagnosis increases after the age of 40 and rises sharply after the age of 50. It is believed that about 5 to 10% of colorectal cancers are the result of genetic factors. Therefore, mutations in genes engaged in the DNA repair pathway like *MLH1* and *MSH2* genes should be mentioned as responsible for hereditary non-polyposis colorectal cancer (HNPCC). On the other hand, mutations in tumor suppressor genes are also associated with the risk of cancer, *e.g.* the *APC* gene is related in familial adenomatous polyposis – FAP (Haggar and Boushey, 2009).

In recent years, the TGFβ signaling pathway, which may act as a tumor suppressor or a promoter depending on the stage of the disease, has become the subject of many studies (Padua and Massagué, 2009; Yang *et al.*, 2010; Heldin *et al.*, 2012). The TGFβ signaling pathway begins with the connection of the ligand, *e.g.* TGFβs, bone morphogenetic proteins (BMPs), growth differentiation factors (GDFs), nodals, activins, and inhibins with the receptor. There are three types of receptors in the cell membrane. The TGFβ receptors type I and type II (defined as TβRI and TβRII) are directly involved in signal transduction (Alarmo *et al.*, 2007; Yang *et al.*, 2010; Olsen *et al.*, 2015). Devoid of enzymatic activity, the TGFβ receptors type III (TβRIII) such as betaglycan regulate the availability of TβRI and TβRII for ligands (Padua and Massagué, 2009). Next to the binding of ligands to TβRII, TβRI is enlisted, transphosphorylated, and activated to phosphorylate the downstream mediators, receptor-regulated SMADs proteins. These transmit the signals to the cell nucleus where the gene transcription is modulated. In addition to the classical pathway, TGFβ signaling pathways independent of SMADs proteins can be distinguished. Also, PI3-kinase, p38 kinase, and small GTPase pathways such as RhoA, PKN, and Rock should be mentioned (Yang *et al.*, 2010; Dean *et al.*, 2017).

TGFβ as a tumor suppressor induces apoptosis or autophagy, inhibits cell cycle, as well as the expression of growth factor, cytokines and chemokines. Many mutations in genes encoding receptors and decreased expression of components of the TGFβ pathway are documented in carcinoma. Additionally, the lack or downregulation of TβRI, TβRII, or SMADs is often associated with a worse prognosis (Yang *et al.*, 2010). TGFβ as a tumor promoter determines mesenchymal-epithelial transition (MET), increases the activity of proteases, decreases immune response, promotes angiogenesis, and modulates the cytoskeletal architecture and extracellular matrix (Padua and Massagué, 2009; Yang *et al.*, 2010; Heldin *et al.*, 2012). Aggressive and rapidly proliferating gliomas have a high TGFβ and SMADs activity. In animal models, an increase of metastasis to other organs was observed after enhancement of TGFβ signaling. The opposite effect, namely a reduction in the ability to metastasize, was observed after suppressed TGFβ transduction (Yang *et al.*, 2010).

In this study, we focused on a gene encoding one of the proteins belonging to the TβRII receptors family (Jung *et al.*, 2004). *ACVR2A* gene also called *ACVR2*, *ACTRII*, or *ACTRIIA* is located on the long arm of chromosome 2 (location 2q22.2-q23.3) and has the overall length of 83.3 kb (Fitzpatrick *et al.*, 2009). The lead role of a protein encoded by this gene is the mediation of activin functions. The activin A receptor type 2A (ACVR2A) is constructed of 513 amino acids, and consists of an extracellular, a transmembrane and a cytoplasmic serine-threonine kinase domains. Except for the ability to transfer phosphate groups, the protein exhibits transferase and tyrosine kinase activity (Jung *et al.*, 2004). Signal transduction begins with the connection of activin to the ACVR2 extracellular domain and the formation of heterodimer complex with ACVR1. Firstly, there is a phosphorylation of ACVR1 and intracellular effector proteins SMAD2 and SMAD3. Phosphorylated SMAD2 and SMAD3 form a complex with SMAD4, which is transported into the cell nucleus to regulate gene transcription (Hempen *et al.*, 2003; Jung *et al.*, 2004).

ACVR2A is believed to be a tumor suppressor that inhibits the growth and differentiation of cells. Its inactivation could lead to the development of colorectal cancer (Ballikaya *et al.*, 2014). For this reason, in the study, the expression of the *ACVR2A* gene in patients with colorectal cancer was estimated. The level of *ACVR2A* expression for age and sex of respondents was assessed. The correlation with the level of *ACVR2A* expression and clinical stage was also determined according to the TNM classification and histological grade of the tumor (G).

## Materials and Methods

### Materials

Tissue specimen of colorectal carcinomas were obtained from the Department of Pathology at the Medical University of Lodz, Poland. All specimens were taken during the colon cancer surgery and diagnosed macroscopically by histopathological examination as a colorectal cancer tissue. Collected tissue samples were frozen in liquid nitrogen immediately after the surgery and then stored at -80 °C until processed for RNA isolation. All experiments were carried out with the license of the local ethics committee (No. RNN/8/08/KE) and patients informed consent.

### RNA extraction

Total RNA was isolated from frozen sections of colorectal cancer tissue using the Total RNA Prep Plus Minicolumn Kit (A&A Biotechnology, Poland) according to the manufacturer’s protocol. The concentration of RNA in samples after isolation was measured using a spectrophotometer. Based on the obtained concentrations, the amount of RNA added to a reverse transcription reaction was determined and standardized in all samples. The isolated RNA has an *A*_260/280_ ratio of 1.8-2.0. Until analysis, RNA samples were stored at -80 °C.

### Reverse transcription

Total RNA isolated from tissue specimens of colorectal cancer was reversely transcribed into complementary DNA (cDNA) in accordance with the High Capacity cDNA Reverse Transcription Kit protocol (Applied Biosystems, USA). The reverse transcription was performed under the following conditions: 25 °C for 10 min, 37 °C for 120 min, and 85 °C for 5 min. The final concentration of RNA used to prepare the reaction mixture was 0.1 μg/μL for each sample. cDNA synthesized by RT reaction was stored at -20 °C until analysis. To check the presence of cDNA in each sample, the PCR amplification of *ACTB* gene was performed (sequences for *ACTB* primer set: forward 5’-GTGGGGCGCCCCAGGCACCA-3’; reverse 5’-CTCCTTAATGTCACGCACGATTTC-3’). *ACTB* gene encoding beta-actin, belonging to the housekeeping genes, was used as a reference. Only the samples that showed the presence of PCR product for *ACTB* gene (540 bp) were selected for further analysis.

### PCR amplification

In the next step of the analysis, a qualitative assessment of *ACVR2A* gene expression was performed. In this analysis an *ACVR2A* gene fragment was amplified by polymerase chain reaction. The PCR reaction was carried out using ready-made solution containing *Taq* DNA polymerase, dNTPs, MgCl_2_ and reaction buffers (Biotool, Germany), according to the manufacturer’s protocol. The 20 μL reaction mixture for PCR consisted of 5 μL of 2x PCR Super Master Mix (Biotool, Germany), 0.7 μL of each primer at a concentration of 0.5 μM (sequences for the *ACVR2A* primer set were: forward 5’-AGGGTTCACTATCAGACTTTC-3’; reverse 5’-GTAAATATGCCAATCCTCTAGC-3’), 1 μL of cDNA template, and distilled water to a final reaction volume of 20 μL. In every experiment, a negative control sample (reaction mixture without cDNA template) was used. The PCR reaction conditions included the initial denaturation for 5 min at 95 °C, 34 cycles consisting of 3 steps (denaturation for 1 min at 95 °C, annealing for 1 min at 57 °C and elongation for 1 min at 72 °C) and final elongation for 7 min at 72 °C. PCR amplifications were carried out in MJ Mini Thermal Cycler (Bio-Rad, USA). The presence of 96 bp PCR products for the *ACVR2A* gene was assessed by electrophoresis in 2% agarose gels. For quantitative assessment, only samples that showed the mRNA expression of *ACVR2A* gene in qualitative analysis were included.

### Real-time PCR

**Figure 1:**
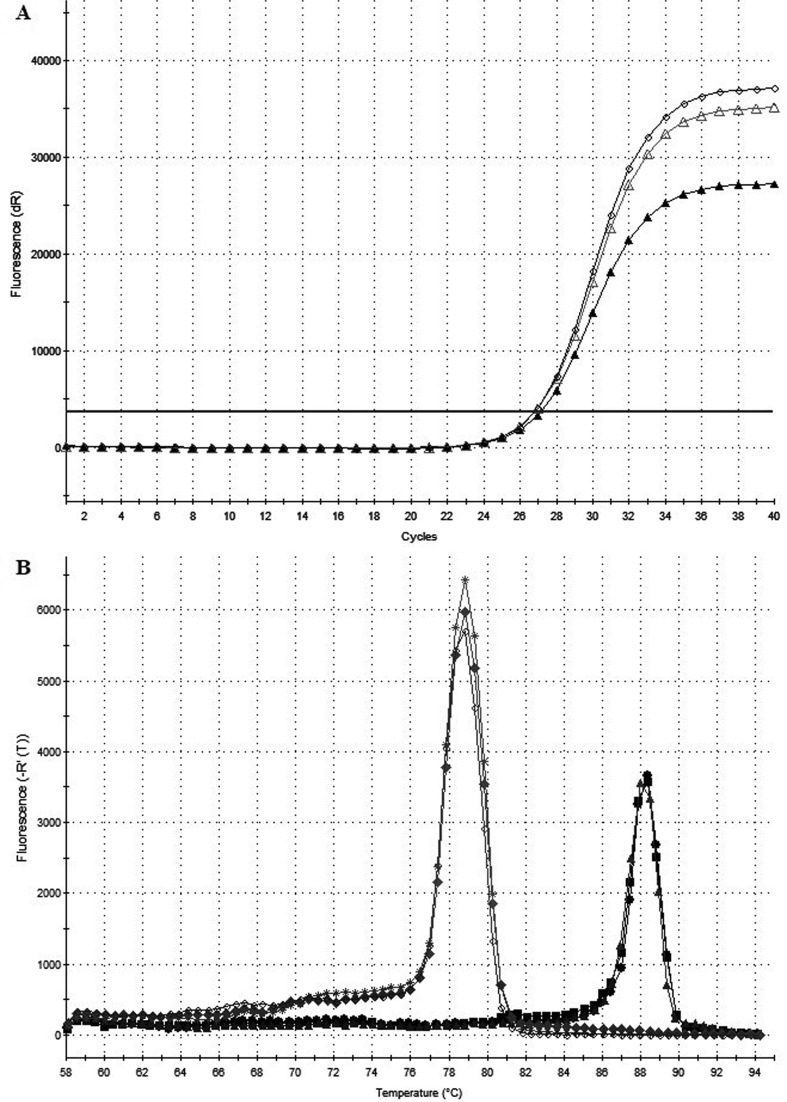
mplification plot of qPCR reaction for samples with fluorescence threshold (A) and melting curve of amplification products (B). The melting temperature (Tm) for the reference gene *ACTB* was 88 °C and for the *ACVR2A* gene was 79 °C. The samples were made in triplicate.

Real-time PCR was used for quantitative assessment of *ACVR2A* (investigated gene) and *ACTB* (reference gene) mRNA expression. Amplification reactions were performed using a MX3005P QPCR SYSTEM (Stratagene, USA) in accordance with the SYBR^®^Green JumpStart TaqReadyMix protocol (Sigma Aldrich, Germany)*.* The reaction mixture for both genes consisted of 7.5 μL of JumpStart Taq ReadyMix (Sigma Aldrich, Germany), 0.7 μL of 10 μM solution of each primer, 1 μL of cDNA template, and distilled water to final reaction volume of 16 μL. The same set of primers of the qualitative analysis was used. All amplification reactions for the two genes were carried out in parallel, in separate tubes and in triplicate for each sample to ensure reproducibility of the reaction. In each experiment, a negative control sample also tested in triplicate was included. The real-time reaction conditions included the initial denaturation step at 95 °C for 10 min and 40 cycles consisting of 3 steps: denaturation at 95 °C for 30 s, annealing at 58 °C for 1 min, and elongation at 72 °C for 1 min. To assess the real-time reaction specificity, melting curve analysis was performed for all amplification products of *ACVR2A* and *ACTB* genes. The obtained Tm (melting temperature) for amplification products of *ACVR2A* was 79 °C and for *ACTB* it was 88 °C (Figure 1). The threshold was manually set at the same level for all analyzed samples to determine Ct values. The mean of the obtained Ct values for both genes was counted. The efficiency of the kinetic PCR reaction was estimated based on the analysis of the standard curves for both genes. The standard curves were obtained by five serial 10-fold dilutions of quantified PCR products. The efficiencies of PCR reactions were calculated from the slopes of the standard curves according to the equation E=10[-1/slope]-1. Because the values of efficiencies for both genes were similar (95% for *ACVR2A*, 93% for *ACTB*), the ΔΔCt method proposed by Livak and Schmittgen was used to calculate the relative level of *ACVR2A* expression. The mean Ct values for the *ACTB* and *ACVR2A* genes obtained for all tested samples were adopted as a calibrator.

### Statistical analysis

The software STATISTICA12 (StatSoft, Inc., 2014) was used for statistical analyses. The collected quantitative data was checked for conformity with a normal distribution using the Shapiro-Wilk test. Due to the lack of conformity with normal distribution a comparative statistical analysis using the nonparametric U-Mann Whitney test was performed. A *p* < 0.05 was assumed as significant in all tests.

## Results

Eighty-four cases of colorectal cancer were qualitatively analyzed to check the *ACVR2A* gene expression. *ACVR2A* gene expression was observed in all tested samples, which were used for further qualitative analysis using the real-time PCR. The statistical analysis included all 84 cases. In the studied group, the expression level of *ACVR2A* relative to *ACTB* was different and ranged from 0.0234 to 85.5424 with a median value 0.786. The detailed clinical description of all patients is shown in Table 1.

**Table 1:** Clinical characteristics of 84 patients with colorectal cancer. The *p*-values were calculated by Mann Whitney test.

Variables	Patients (n = 84)	*p-*value
Sex:		
male	37	0.172
female	47	
Age (years):	63 (34-82)	0.774
Tumor localization:		
cecum or colon	53	0.051
rectum	27	
Type of tumor:		
tubular	59	0.375
mucinous	10	
Histological grading:		
G1 or G2	59	0.692
G3	24	
TNM staging:		
I or II	46	0.261
III or IV	38	
Size and depth of primary tumor invasion (T):		
pT1 or pT2	22	0.04
pT3 or pT4	62	
Presence of metastases in the regional lymph nodes (N):		
N0	48	0.439
N1 or N2	31	
Distant metastases (M):		
presence of metastases	14	0.782
lack of metastases	68	
Lymphocytes in tumor tissue:		
presence of lymphocytes	37	0.179
lack of lymphocytes	47	
Vessel invasion:		
presence of vascular invasion	53	0.219
lack of vascular invasion	31	

The studied population consisted of 47 women and 37 men. Patients age was between 34 and 82 years, with the median age at the time of diagnosis of 63 years. The analyzed population was divided into two groups according to age. The first group included the 38 persons aged between 34 and 60 years old and the second group consisted of 46 people aged over 60 years. No statistical difference was observed between *ACVR2A* gene expression level and patients’ gender (*p*=0.172) or age (*p*=0.774).

In 53 of the cases, the tumors were located in the cecum or colon, the other 27 tumors were located in the rectum. Although a higher relative level of *ACVR2A* gene expression was observed in the group of patients with tumors located in the rectum, this difference was not statistically significant (*p*=0.051). All examined tumors were histologically classified as adenocarcinomas. The tubular type of tumor was predominant in the studied group (59 cases had a tubular type and 10 cases had a mucinous type). No correlation was found between tumor histological and the expression of the investigated gene (*p*=0.375).

Due to the histological grading, most tumors were classified as G1 or G2 grade (59 cases); the other 24 tumors had a G3 grade. In this study, cases were divided into 2 groups: cancers that were of low or intermediate grade (group 1) and of high grade (group 2). Comparison of *ACVR2A* expression level with histological grading between the groups did not show a statistical dependence (*p*=0.692).

The *ACVR2A* gene expression level was compared with several clinicopathological parameters, such as the size and depth of primary tumor invasion (T), the presence of metastases in the regional lymph nodes (N), and the presence of distant metastases (M) according to the TNM classification. No dependence was observed when comparing *ACVR2A* gene expression with the lymph nodes and distant metastases (*p*=0.439, *p*=0.782 respectively for N and M). On the other hand, in cases with more advanced tumors (pT3 or pT4 wall depth penetration), the relative level of *ACVR2A* gene expression was higher than in cases with lower tumor invasion (pT1-pT2). This difference was statistically significant (*p*=0.04). In order to confirm that the expression level of the investigated gene was correlated with the stage of cancer, another evaluated parameter was pTNM staging. Based on this cancer staging method, the studied population was grouped into less advanced (46 cancers with stages I or II) and more advanced cases (38 cancers with stages III or IV). Comparison of *ACVR2A* gene expression level with pTNM staging between the groups did not show a significant dependence (*p*=0.261).

The presence of lymphocytes in tumor tissue is a positive prognostic indicator in advanced colon cancer. Therefore, in another analysis, the *ACVR2A* gene expression was compared with the presence of tumor-infiltrating lymphocytes. In 37 cases, lymphocytes were present in tumor tissue, while in 47 cases they were not. A lower level of *ACVR2A* expression was observed in patients with lymphocytic infiltration. We found that the *ACVR2A* expression level was not significantly related to the presence of lymphocytes (*p*=0.179).

The last analyzed parameter was the presence of vessel invasion, which is a poor prognosis factor in cancers. Vascular invasion was present in 53 cases and in this group of patients the relative level of *ACVR2A* gene expression was higher. Despite this, no association was observed between the level of *ACVR2A* expression and presence of vessel invasion (*p*=0.219).

## Discussion

Numerous screening tests implemented in recent years have reduced mortality and incidence of colon cancer in the world. Nevertheless, the five-year survival remains low due to metastasis (Bauer *et al.*, 2015). The process of metastasis is associated with reduced or transformed TGFβ susceptibility and increased expression or activation of the TGFβ ligands. Many studies were conducted to clarify the transition mechanism of the TGFβ signaling pathway from a tumor suppressor to promoter and as a result, two signaling SMAD-dependent and SMAD-independent pathways have been extracted. It is believed that both of them contribute to the development of cancer (Yang *et al.*, 2010).

ACVR2A is a ligand of activin A protein, which is an important regulator of pregnancy. Activin A occurs physiologically, *e.g.*, in the endometrium, placenta and vascular endothelium and regulates the remodeling of the uterine spiral arteries, which are reduced in preeclampsia. The elevated level of activin A in the serum of women with preeclampsia has been documented and for this reason is considered as a preeclampsia marker (Fitzpatrick *et al.*, 2009). In addition to the *ACVR2A* mutations in preeclampsia, vascular dysfunction leading to hypertension, proteinuria, or edema are reported. Many sources also indicate the potential impact of *ACVR2A* gene mutations on multiple synostoses syndrome (Jung *et al.*, 2004; Fitzpatrick *et al.*, 2009; Van Dijk and Oudejans, 2011). Wang *et al.* (2014) demonstrated that the ACVR2A protein level correlates with the severity of sepsis. The contribution of *ACVR2A* has also been confirmed in the gastrulation, spermatocytogenesis and spermiogenesis processes ([Bibr B26] [Bibr B27] AmiGO).

*ACVR2A* is involved in important signaling pathways, for example PEDF-induced signaling, TFG-β signaling pathway, or signaling pathways regulating the pluripotency of stem cells, which may be related to the initiation of the carcinogenesis process (PathCards). Among others things, the association of the *ACVR2A* gene mutation with the development of prostate and lung cancer has been confirmed (Rossi *et al.*, 2005; Dean *et al.*, 2017).

**Figure 2:**
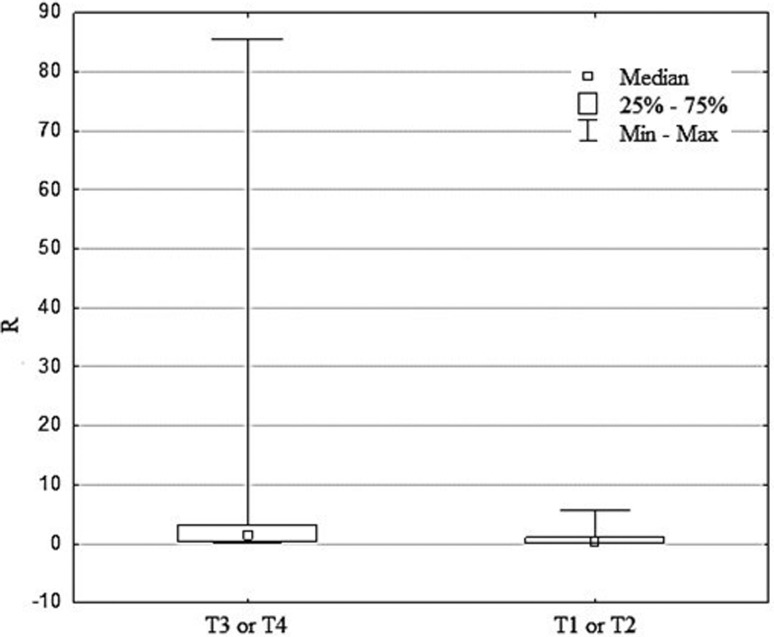
Comparison of *ACVR2A* expression level (R) with size and depth of primary tumor invasion (T) according to TNM classification.

In this study, the level of mRNA were compared with the clinical stage of cancer (the TNM Classification of Malignant Tumors) and its components. There was no statistically significant association between *ACVR2A* gene expression and the presence of metastases in the regional lymph nodes (*p*=0.439) and distant metastases (*p*=0.782). A similar result was achieved by evaluating the level of *ACVR2A* gene expression with the TNM staging (*p*=0.261). One of the TNM classification components, namely the size and depth of primary tumor invasion, deserves more attention. After dividing the study cohort into two groups, it appeared that the more extensive tumor group (T3 or T4) had higher *ACVR2A* gene expression compared to the less advanced tumor group (T1 or T2). This difference was statistically significant (*p*=0.04) and shown in Figure 2.

In an investigation, Jung *et al.* (2006) tested the effects of *TGFBR2*, *BAX*, and *ACVR2* mutations according to tumor stage, grade, and size in MSI-H colon cancer. They found that poor histological grade and larger tumor volume were correlated with mutant *ACVR2*. In this experiment, it was observed that the majority of frameshift mutations were biallelic and lead to the loss of protein expression. Activin signaling is believed to be a growth suppressor and inhibitor of this signaling by mutated *ACVR2A*, and loss of protein expression may increase tumor growth (Deacu *et al.*, 2004; Jung *et al.*, 2006). In addition, the restoration of ACVR2A activity was seen to inhibit the growth of colon cancer cells (Deacu *et al.*, 2004; Chung *et al.*, 2008). This fact was explained by the increase of *de novo* synthesis of proteins as CALU, IBP2, LETM1, PRS8, SF3B3, and TNPO1 responsible for growth inhibition and induction of apoptosis (Ballikaya *et al.*, 2014). In many studies, efforts have been made to restore the activation pathway via drugs or miRNAs. For instance, Fuchs *et al.* (2014) noted that miR-27 inhibits TGFβ signaling pathway genes, including *ACVR2A* (Fuchs *et al.*, 2014). In a mouse model, *acvr2a* was also found to be regulated by miR-29b and miR-181a (Plank *et al.*, 2015). Attempts have also been made to reduce the mutations in the *ACVR2A* gene by mesalazine. As reported by Campregher *et al.* (2010), 5-ASA reduces microsatellite instability and improves replication fidelity in repeat sequences of *TGFBR2* and *ACVR2A* genes. Hence, taking into consideration the potential role of the TGFβ signaling pathway genes in metastases, the use of suitable drugs or miRNA may be helpful in the future to reduce the expression of these genes and avoid metastases in people at risk (Campregher *et al.*, 2010; Fuchs *et al.*, 2014).

The study by Jung *et al.* (2006) found in two cohorts of 172 and 503 patients a different incidence of *ACVR2A* mutations (4.5% vs 32.5% respectively), indicating a high variability of mutations in populations. In another study, the *ACVR2A* gene was seen mutated in 90.9% of MSI colorectal cancer cases (Pinheiro *et al.*, 2015). In later a subsequent study by Jung *et al.* (2009), in which 51 cases of MSS colon cancers were examined, loss of *ACVR2A* expression was observed in only 14% of cases, and in an immunohistochemistry study conducted by Jung *et al.* (2004), the loss of *ACVR2A* expression was observed in 62% of MSI colorectal neoplasms and protein presence was observed in all MSS tumors. The presence of protein in all our samples might suggest that they originated from MSS colon cancers, but this must be determined at the protein level.

In addition, discrepancies may have resulted from a relatively small number of colorectal cases in our study (84 patients were surveyed) and minor differences within the study group in case of cancer invasion and metastasis. For a complete view of *ACVR2A* gene expression in different stages of cancer development it seems advisable to extend the study to include patients with colorectal cancer to compare *ACVR2A* expression in each of the T1, T2, T3, and T4 groups separately.

Our study apparently contradicts the results obtained by Jung *et al.* (2006), because more advanced tumors (according to the TNM classification) presented higher *ACVR2A* expression. Similarly, overexpression of the *ACVR2A* gene was found in multiple myeloma (Grcevic *et al.*, 2010). In addition, ovarian cancer patients with higher *ACVR2A* expression had shorter disease-free survival compared to those with low expression (Dean *et al.*, 2017).

Inconsistent results were found in other studies of primary tumor size and ACVR2A protein expression. It is possible that despite the high expression, the resulting post-translational protein is dysfunctional and deprived of activity. The phenotypic effect of these lesions may be a greater tumor size. Further research is needed to analyze the functionality of the resulting protein by proteomics techniques.
